# The Assessment of a Personalized Nutrition Tool (eNutri) in Germany: Pilot Study on Usability Metrics and Users’ Experiences

**DOI:** 10.2196/34497

**Published:** 2022-08-04

**Authors:** Birgit Kaiser, Tamara Stelzl, Paul Finglas, Kurt Gedrich

**Affiliations:** 1 Research Group Public Health Nutrition ZIEL - Institute for Food & Health TUM School of Life Sciences, Technical University of Munich Freising Germany; 2 Chair of Analytical Food Chemistry TUM School of Life Sciences, Technical University of Munich Freising Germany; 3 Quadram Bioscience Institute Norwich United Kingdom

**Keywords:** Food Frequency Questionnaire, dietary assessment, Diet Quality Score, web application, digital nutrition, personalized nutrition, system usability, internet, eNutri, EIT Food Quisper

## Abstract

**Background:**

To address the epidemic burden of diet-related diseases, adequate dietary intake assessments are needed to determine the actual nutrition intake of a population. In this context, the eNutri web app has been developed, providing online automated personalized dietary advice, based on nutritional information recorded via an integrated and validated food frequency questionnaire (FFQ). Originally developed for a British population and their dietary habits, the eNutri tool has specifically been adapted to the German population, taking into account national eating habits and dietary recommendations.

**Objective:**

The primary aim of this study is to evaluate the system usability and users’ experience and feedback on the eNutri app in a small-scale preliminary study. The secondary aim is to investigate the efficacy of personalized nutrition (PN) recommendations versus general dietary advice in altering eating habits.

**Methods:**

The app was piloted for 4 weeks by 106 participants from across Germany divided into a PN group and a control group. The groups differed according to the degree of personalization of dietary recommendations obtained.

**Results:**

An overall System Usability Scale (SUS) score of 78.4 (SD 12.2) was yielded, indicating an above average user experience. Mean completion time of the FFQ was 26.7 minutes (SD 10.6 minutes). Across subgroups (age, sex, device screen sizes) no differences in SUS or completion time were found, indicating an equal performance for all users independent of the assigned experimental group. Participants’ feedback highlighted the need for more personalized dietary advice for controls, while personalized nutritional recommendations improved the awareness of healthy eating behavior. Further improvements to the eNutri app were suggested by the app users.

**Conclusions:**

In total, the eNutri app has proven to be a suitable instrument to capture the dietary habits of a German population sample. Regarding functionality, system usability, and handling, direct user feedback was quite positive. Nutritional advice given was rated ambivalent, pointing to several weaknesses in the eNutri app, minimizing the system’s full potential. A higher level of personalization within nutritional advice subjectively improved the app’s usability. The insights gained will be used as a basis to further develop and improve this digital diet assessment tool.

## Introduction

Unhealthy diets and their consequences on health are still a matter of high relevance, especially regarding noncommunicable diseases such as diabetes, cardiovascular disorders, or cancer. According to the latest Global Burden of Disease Study [1], dietary risk factors (eg, high sodium intake, low consumption of whole grains and fruits) globally accounted for around 11 million deaths and 255 million disability-adjusted life-years in 2017. With the advent of the internet and computerization, digital applications are increasingly replacing traditional pen/paper methods for collecting nutritional data. Web-based tools were found to collect data of a similar quality compared with their handwritten origins and are preferentially used by younger populations [2].

In this context, *information and communications technologies* (ICTs), such as web-/computer-based services as well as mobile phones, are used to record dietary behavior. Data about nutritional intake can be captured passively through sensing or tracking techniques as well as actively by, for example, manual data entry [3]. Regarding (computerized) instruments, food frequency questionnaires (FFQs) are one of the most commonly used tools to track and assess dietary habits of individuals. Based on a preselected list of foods, individuals report the frequency of their habitual dietary intake. In general, consumption data are collected retrospectively over a recall period of a few weeks up to 1 year [4]. Various types of ICT solutions such as smartphone or tablets and desktops or laptops have been shown to be eligible for the application of digital FFQs [5-7]. The integration of dietary assessment tools into ICTs provides an unobstructive way to offer nutritional advice, which is comparable to nutritional guidance provided by nutrition professionals [8]. Advanced technologies are known to drive healthy changes in dietary intake [9], but to promote long-term use of digital health technologies and behavior change, focus should be placed on user experience and content [3]. Time-consuming data entry and poor overall user interface negatively affect users’ experience [10], whereas inclusion of energy and macronutrient content are promoting factors for user system interaction [11].

ICT apps capable of providing personal nutritional advice have attracted considerable attention in recent years [12]. To date, very few apps are equipped with the necessary decision engine for generating automated dietary recommendations in a personalized format that are also valid [13]. To address this need, the eNutri web app has been developed at the University of Reading (Hugh Sinclair Unit of Human Nutrition, United Kingdom) to provide automated personalized nutrition (PN) recommendations with a high degree of personalization [13]. As part of this approach, a validated web-based graphical FFQ illustrating different portion sizes of food items has been integrated into the eNutri app. The app provides personalized dietary recommendations based on retrospectively collected data of users’ food consumption habits, considering individual dietary preferences, BMI, sex, and possible dietary restrictions (eg, abstaining from meat consumption). In addition to personalized advice (by nutrition experts) [14], the app can show generic dietary recommendations based on national guidelines with a low level of personalization [15]. Results of the evaluation of the eNutri app suggest a good usability and acceptance for the online dietary intake assessment [16]. In collaboration with the University of Reading, a German version of the eNutri app was developed, which was adapted to German food consumption habits and underwent some minor modifications (eg, translation into German language, data protection adjustments, replacement of the UK food and nutrient database of McCance and Widdowson [17] by the German Food Code and Nutrient Database or BLS [18]). The dietary recommender system in the app was acquired from the British research partners without modification. In a subsequent 4-week pilot study (eNutri2019 study), the app was field tested in a German population sample [19]. The study was part of the European Institute of Innovation and Technology (EIT) Food Quisper (Quality Information Services and Dietary Advice for Personalized Nutrition in Europe) Project, which aims to create a digital platform for evidence-based PN services and data [20].

In this study, the primary objectives were to assess the eNutri app’s suitability for use in a healthy adult population in Germany and to gather user feedback regarding its usability and content. An additional aim was to identify the scope for improvement based on the users’ feedback data. Furthermore, this study aimed to evaluate the effectiveness of PN recommendations over generic dietary advice.

## Methods

### Study (eNutri2019) Design

The German eNutri app was applied within the eNutri2019 pilot study, which was conducted in November and December 2019 in a German population sample. A subsample of the overall study results has been published earlier, comparing specifically the dietary behavior of female vegetarians with omnivores [19]. The analyses contained within this report refer to all study participants (106 participants overall) and are not limited to a selected subset. Participants’ dietary intake was assessed retrospectively at 2 different time points, first at baseline (time point 1 [t1]) and second after 4 weeks at the end of the study (time point 2 [t2]). After the baseline survey, study participants were provided with dietary recommendations either in a personalized or in a generic format, according to the group (PN or control) they were assigned to.

### Data Collection

#### Overview

To participate in the eNutri2019 pilot study, individuals had to register online. After passing the inclusion criteria, participants were granted access to the eNutri app via an anonymous alias email address to ensure protection of privacy. Dietary intake, physical activity, anthropometrics, device information, system usability, and feedback were gathered within the eNutri app. Data input was requested at t1 and t2. Participants received reminders via email on dietary recommendations provided after the first survey and to announce the second survey date.

#### Anthropometric and Sociodemographic Data Collection

For anthropometric measurements, study participants were provided with step-by-step instructions to accurately measure their body height and weight by themselves. Self-reported sex, body height, and weight were gathered within the eNutri app, which automatically calculated the BMI (kg/m^2^) as the weight-to-height ratio. Both sex and body height were recorded at t1, whereas only body weight was captured at both time points.

#### Dietary Assessment

At time points t1 and t2, dietary intake of the previous 564 weeks was assessed retrospectively via a self-administered FFQ integrated in the eNutri app. Before starting the survey, participants received guidance through a tutorial on how to correctly complete the FFQ within the app, which they could return to at any time. The food list and portion sizes used for the FFQ were based on the validated questionnaire of the Food4Me study, a pan-European randomized controlled dietary intervention study [21]. The food list was adapted regarding country-specific popular German food items (eg, pretzels, rusk, fruit nectar, sweet egg dishes). The final FFQ comprised 156 food items. Foods and respective portion sizes were presented as photos, based on representative servings as defined in the BLS [18]. For each food item, a total of 7 different portion sizes were displayed, 3 of which were illustrated with photos, plus arrow buttons on either side of each image to select smaller/larger portion sizes than the ones depicted. Intake frequencies were determined based on 9 different options to choose from (<1/month, 1-3/month, 1/week, 2-4/ week, 5-6/week, 1/day, 2-4 /day, 5-6/day, or ≥7/day). An illustrative example of the eNutri input and output visualization module is provided by Fallaize et al [22]. Energy and nutrient intake values were calculated automatically by the system in reference to the BLS.

#### Diet Quality Score

The nutritional value of the reported dietary habits was quantified using an 11-item Diet Quality Score (DQS) developed for a Western European population [22]. Each individual’s DQS was calculated from the eNutri FFQ data at t1 and t2. The scoring system was developed and validated by the University of Reading, based on data from the EPIC (European Prospective Investigation into Cancer) Norfolk cohort study. It is calculated based on 7 food components scoring positively (vegetables; fruit; wholegrain products; healthy fats; oily fish; nuts and seeds; and pulses), and 4 scoring negatively (free sugar, salt, alcohol, and red/processed meat). Scores of all components contributed equally to the overall DQS (interval 0-110). The DQS was reported to be predictive of cardiovascular disease, inflammatory heart disease, acute myocardial infection, and all-cause mortality risk reduction [15].

#### Dietary Recommendations (System Feedback)

Data collected from study participants were used to generate personalized nutritional feedback. The self-reported data provided by a user (ie, sex, weight, height, food intake, and frequency) are processed to compute an individual’s BMI and DQS, and an integrated decision engine (algorithm) calculates a “healthiness score” for each FFQ item, equivalent to which foods/drinks would have the greatest/worst impact on the DQS if an additional portion per day would be consumed. This score is translated into dietary recommendations within the app, visualized in 5 output sections, namely, “foods to boost,” “foods to try,” “foods to reduce,” “foods to keep eating,” and “foods to keep avoiding.” A more detailed description of the recommender system of the eNutri app is provided by Fallaize et al [22]. A *tip* section featuring explanations and background information on the specific food recommendation was integrated into the dietary feedback to support users to implement their food-based recommendations into their dietary habits.

Aiming to assess the effectiveness of the personalized dietary advice, participants were randomly assigned to either the control or the PN group, according to their sex, BMI, and age classification, using the method of minimization [23]. After completion of the baseline questionnaires, participants in the control group received generic population-based nutritional recommendations based on national dietary guidelines (German Nutrition Society [DGE]) [24]. The participants assigned to the PN group were provided with personalized dietary feedback, based on their individual FFQ responses and their stated food preferences [22]. After the administration of second questionnaire at t2, both groups received personalized dietary feedback.

#### System Recording of Time Stamps and Device Screen Size

As a background process, the system automatically recorded time stamps, including the date and the start and end time of FFQ processing. A record was also made of whether a questionnaire was completed in full or only in part. Completion time was calculated based on collected time stamps (for FFQ, system usability and feedback items), which were recorded upon user activity within the app for the first survey (t1). Information on screen sizes was collected as part of the browser details. Device screen sizes were categorized into 3 groups: small <480 pixels, medium 480-1240 pixels, and large >1240 pixels [16].

#### System Usability Scale and Participants’ Feedback

For assessing the efficiency, effectiveness, and satisfaction of app usage [25], the overall System Usability Scale (SUS) score achieved was displayed as a graphical progress bar with corresponding numeric values within the eNutri app after completion of the baseline FFQ. The underlying SUS questionnaire surveyed 10 items in total, addressing the app’s usability based on statements such as “I found the system unnecessarily complex” [26]. Each item provides 5 response options ranging from “strongly agree” (equal to 5 points) to “strongly disagree.” (equal to 1 point). The qualitative metrics are converted into a numerical scale yielding a total score between 0 and 100 points. Scores higher than 68 points are considered as above average [27,28]. It has been shown that the SUS score is positively correlated with user acceptance [28].

Additional questions were displayed after completing the second eNutri FFQ at t2. Participants were provided with a series of 5-level Likert scale and multiple-choice questions, as well as with a free-text question regarding their subjective feedback on the eNutri app. Feedback questions were focused on the overall user-friendliness of the eNutri app, the impact of the app on perceptions of a healthy dietary behavior, changes in dietary intake due to the app intervention, and the evaluation of the (dietary) recommendations. Furthermore, participants were asked to rate the user-friendliness of the eNutri app, as well as the app in its entirety, according to a 5-star rating system as commonly applied in app stores (eg, Google Play or iTunes), with 5 stars for the highest and 1 star for the worst rating. Willingness to pay was queried based on several preset pricing options to choose from (€0.00, €0.50 [US $0.53], €1.00 [US $1.07], €2.50 [US $2.67], €5.00 [US $5.34], and >€5.00 [>US $5.34]).

### Statistical Analysis

The statistical analysis was directed toward usability metrics, where we focused on SUS scores and feedback questions. Furthermore, subgroups were defined by age, sex, and device screen sizes to compare the usability of the eNutri app across different user groups. Feedback questions were analyzed with respect to participant’s group assignment (PN and control). Categorial answer options were transformed into numerical answer options with numerical gradation from 1=strongly disagree to 5=strongly agree. Likert-scale coded data and data from the feedback questionnaire at t2 were analyzed by applying nonparametric tests (chi-square test, Fisher exact test, and Mann–Whitney *U* test). Parametric tests (unpaired *t* test) were applied to check for statistical difference between subgroups and SUS analyses. In addition, written feedback on free-text questions was summarized and then categorized into main topics. Statistical data analysis was performed using Microsoft Excel 2016 (Microsoft Corporation) and R 3.6.0 (R Foundation). *P* values ≤.05 were considered statistically significant.

### Ethics Approval

Ethical approval was granted by the Research Ethics Committee of the Technical University of Munich (approval no. 328/19S).

## Results

### Study Population

A total of 792 potential participants registered for the eNutri2019 pilot study (Multimedia Appendix 1), among which 297 registrants were found eligible. Of these, 167 study participants created an account within the eNutri app and 158 completed the first FFQ; 4 participants actively withdrew from the study and 29 were lost to follow-up. After data cleaning (removing missing, imprecise, or implausible information, such as BMI >60 kg/m^2^ or <15 kg/m^2^; total energy intakes <600 kcal/day or >4500 kcal/day), a total of 106 participants remained. The sociodemographic characteristics of the study participants included in the data analysis are listed in Table 1.

**Table 1:** Sociodemographic characteristics of study participants and selected outcome parameters.

Participant characteristics		Total (N=106), n (%)	Total, mean (SD)	PN^a^ group (n=53), n (%)	PN group, mean (SD)	Control group (n=53), n (%)	Control group, mean (SD)
**Sex**							
	Female	92 (86.7)	N/A^b^	46 (86.8)	N/A	46 (86.8)	N/A
	Male	14 (13.2)	N/A	7 (13.2)	N/A	7 (13.2)	N/A
**Age**							
	Younger (<40 years)	93 (87.7)	23.4 (4.5)	46 (86.8)	23.5 (4.2)	47 (88.7)	23.3 (4.8)
	Older (≥40 years)	13 (12.3)	51.2 (5.7)	7 (13.2)	52.1 (6.6)	6 (11.3)	50.0 (4.7)
**Level of education**							
	Less than secondary school	6 (5.7)	N/A	4 (7.5)	N/A	2 (3.8)	N/A
	Secondary school	56 (52.8)	N/A	26 (49.1)	N/A	30 (56.6)	N/A
	Completed apprenticeship	2 (1.9)	N/A	2 (3.8)	N/A	0 (0)	N/A
	University degree	42 (39.6)	N/A	21 (39.6)	N/A	21 (39.6)	N/A
**BMI (kg/m^2^)**							
	Underweight (<18.5)	5 (4.7)	16.5 (1.0)	2 (3.8)	16.7 (1.6)	3 (5.7)	16.4 (0.8)
	Normal weight (18.5-24.9)	79 (74.5)	21.7 (1.8)	40 (75.5)	21.9 (1.8)	39 (73.6)	21.4 (1.7)
	Overweight (≥25.0)	22 (20.8)	28.82 (3.58)	11 (20.8)	29.53 (3.84)	11 (20.8)	28.1 (3.3)

^a^PN: personalized nutrition.

^b^N/A: not applicable.

### Evaluation of the Systems Usability

The first survey (FFQ and SUS questionnaire) did not differ between both groups except for the dietary recommendations, which were displayed only at the end of the first survey, after all data were collected from the participants. The mean SUS across all 106 study participants was 78.4 (SD 12.2). PN participants evaluated the eNutri app (mean SUS 79.9, SD 12.5) insignificantly higher than control participants (mean SUS 76.9, SD 11.9; *P*=.14). Female participants had a higher mean SUS score of 78.7 (SD 12.1) compared with male participants, who had a mean score of 76.4 (SD 13.5; *P*=.59). Regarding age groups, younger participants (<40 years) recorded a higher mean SUS of 78.8 (SD 12.0) than older participants (≥40 years), who recorded 75.2 (SD 13.5; *P*=.39). Additionally, SUS scores were considered with respect to the screen size of the device participants used to complete the surveys. Participants with small screen sizes (29/106) rated the app best with a mean SUS of 79.4 (SD 12.3), followed by those with medium-size device screen (7/106) with a mean SUS of 78.9 (SD 13.1) and those with large screen size (70/106), who had the lowest mean SUS of 77.9 (SD 12.2; *P*=.85). Across all subgroups, the mean SUS was greater than 68, indicating a good usability.

### Analysis of Average Survey Completion Times

Completion time could not be assessed for all participants, due to repeated log-ins by some participants. Thus, completion time was analyzed only for 87 participants, who exhibited an average duration of 26.67 minutes (SD 10.6 minutes). Participants of the control group entered their data (mean completion time of 23.81 minutes, SD 7.8 minutes) faster than those of the PN group (mean completion time of 29.45 minutes, SD 12.3 minutes; *P*=.05). Female participants (75/87, 86%) completed the survey faster, with a mean completion time of 25.37 minutes (SD 9.2 minutes), than male participants (12/87, 14%), with a mean completion time of 34.75 minutes (SD 15.4 minutes; *P*=.05). Younger participants (77/87, 89%) had a mean completion time of 25.84 minutes (SD 10.2 minutes) compared with older participants (10/87, 11%), who had a mean completion time of 33.00 minutes (SD 12.42 minutes; *P*=.07). Regarding screen sizes, participants using a medium device screen size for answering the questions (7/87, 8%) were fastest with a mean completion time of 22.86 minutes (SD 7.4 minutes), followed by participants with small screen sizes (20/87, 23%), who had a mean completion time of 25.65 minutes (SD 8.9 minutes). Participants with large screen sizes (60/87, 69%) took longest, with a mean completion time of 27.45 minutes (SD 11.4 minutes; *P*=.76).

### Evaluation of the System’s User-Friendliness

**Figure 1:**
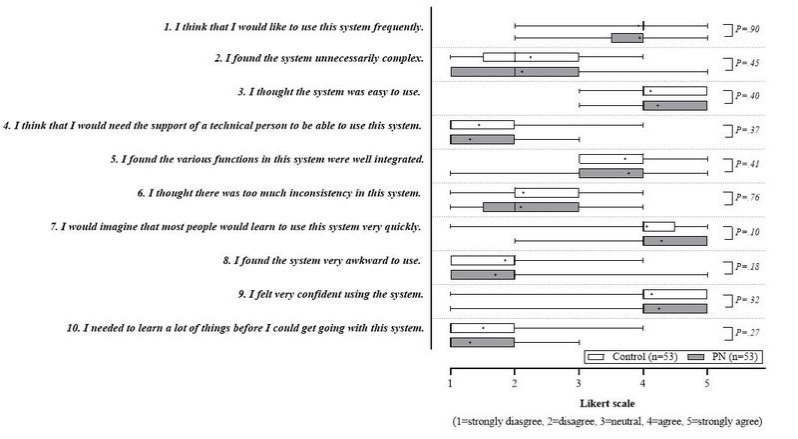
Box plot analysis of the Likert-scale ratings.

To assess participant’s perception of the user-friendliness of the eNutri app, a series of Likert-scale questions were asked at t1, after completion of the first FFQ and before displaying the first dietary report (Figure 1).

Overall, very high Likert-scale scores (above 4; equivalent to very high agreement rates among the study participants) were recorded for the statements regarding “ease of use of the system,” the “rapid learnability” of the system, and the “confidence in using the system” (all with a median of 4.0 for control/PN). Lowest Likert-scale scores (below 2; equivalent to strong or medium disagreement) were detected for the “need of technical support” to use the system, the “need to learn a lot of things before using the system” (both with a median of 1.0 for control/PN), and the “operation awkwardness” of the app (median of 2.0 for control/PN). Spanning from neutral to agreeing, and at a median of 4.0, feedback on the statements “to use the system frequently” and its “well integration” leveled off. For the statements concerning “system complexity and inconsistency,” the interquartile ranges were between 1 and 3 on the Likert scale with a median of 2.0 each.

### Participants’ Feedback On System Usability

**Figure 2:**
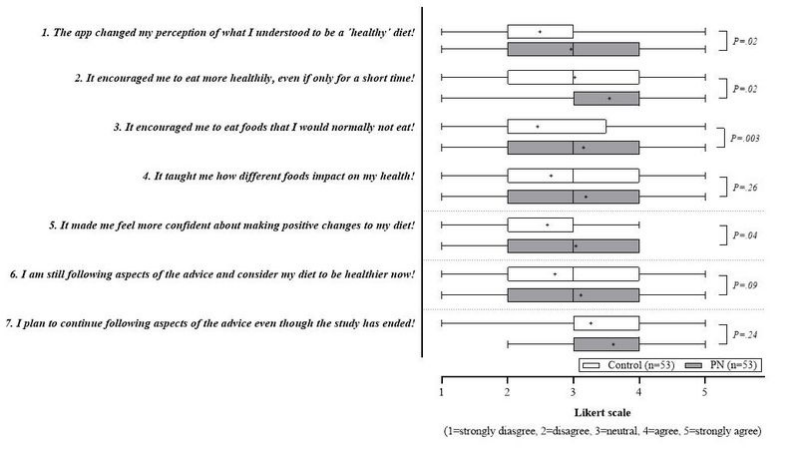
In-app feedback related to eNutri-induced changes in dietary behaviour.

An extended feedback collection conducted after completion of the survey at t2 and after transmission of personalized dietary reports to both PN and control groups (Figure 2) was aimed at providing information about participants’ reflections on their own diets or dietary habits.

The distribution of responses to the various survey statements revealed that, with all interquartile ranges spanning between Likert scale scores of 2 and 4, a significantly higher proportion of study participants in the PN group agreed that the eNutri “encouraged them to eat more healthily, even if only for a short period of time” (median control 3.0; median PN 4.0; *P*=.02). Greater deviation in feedback was further observed for the statement “the app changed their perception of what is a ‘healthy’ diet.” With a median of 2.0, most participants in the control group disagreed, while significantly (*P*=.02) more respondents in the PN group (median 3.0) stayed neutral or agreed. A similar tendency was observed for the statements that the app “encouraged me to eat foods that I would normally not eat” (median control 2.0; median PN 3.0), and “It made me feel more confident about making positive changes to my diet” (median control and median PN 3.0). Referring to the statements “It taught me how different foods impact on my health,” and “I am still following aspects of the advice and consider my diet to be healthier now,” the ratings were quite identical in both groups (median control and median PN 3.0). When asked if participants “plan to continue following aspects of the device though the study has ended,” the median was 4.0 for both groups, signaling that the vast majority of study participants agree.

Regarding the question if participants would “recommend the eNutri app to their family and friends,” on average 35.8% (38/106) of all participants indicated that would *likely* or *highly likely* recommend the app, with same response frequencies in both PN and control groups. Furthermore, participants were asked “What are the reasons for not following your dietary recommendations?” (Table 2), and the most frequently chosen option was “I did not like the recommended food” (total: 30/106, 28.3%; control: 6/53, 11%; PN: 24/53, 45%; *P*<.001). This was followed by the response “I lacked ideas for including the recommended food into my diet” (total: 29/106, 27.4%; control: 17/53, 32%; PN: 12/53, 23%; *P*=.28) and “recommended foods did not fit into my usual meal plans/recipes” (total: 25/106, 23.6%; control: 10/53, 19%; PN: 15/53, 28%; *P*=.25). In total, 24.5% (26/106) of all participants indicated that “other people shop and cook for me” (control: 11/53, 21%; PN: 15/53, 28%; *P*=.37), while on average 13.2% (14/106) responded that they “will not change certain aspects of their diet, regardless of the advice” (control: 4/53, 8%; PN: 10/53, 19%; *P*=.09). Nearly 10.4% (11/106) of all study participants disagreed with the dietary recommendations provided by the eNutri app because they were incompatible with their dietary restrictions. It is also worth mentioning that on average 13% (14/106; *P*=.01) of the study population indicated that “the recommended foods were expensive,” of which almost 21% (11/53) belonged to the PN group. The overall mean star rating of the eNutri app reached 3.3 stars (SD 0.9) out of 5.0. Participants in the PN group rated the eNutri app insignificantly higher (mean stars 3.4, SD 0.9), compared with those in the control group (mean stars 3.2, SD 0.9; *P*=.27).

**Table 2:** Feedback on dietary recommendations provided by eNutri2019 study participants (N=106).

Feedback on nonadherence to dietary recommendations		Control group (n=53), n (%)	PN^a^ group (n=53), n (%)	*P* value
**What are the reasons for not following your dietary recommendations?**				
	I did not like the recommended food	6 (11)	24 (45)	<.001
	I lacked ideas for including the recommended food into my diet	17 (32)	12 (23)	.28
	The recommended foods did not fit into my usual meal plans	10 (19)	15 (28)	.25
	Other people shop and cook for me	11 (21)	15 (28)	.37
	I will not change certain aspects of my diet, regardless of the advice	4 (8)	10 (19)	.09
	I did not agree that the advice would result in a healthier diet for me	11 (21)	9 (17)	.62
	I was not willing to try new foods	1 (2)	1 (2)	.99
	The recommended foods were expensive	2 (4)	1 (21)	.01
	I did not know what to eat instead when replacing less healthy foods	4 (8)	2 (4)	.68
	My dietary restrictions were not considered	3 (6)	2 (4)	.99
	The health benefits of making these changes were unclear	2 (4)	2 (4)	.99
	I was concerned my weight would change	0 (0)	2 (4)	.50

^a^PN: personalized nutrition.

**Figure 3:**
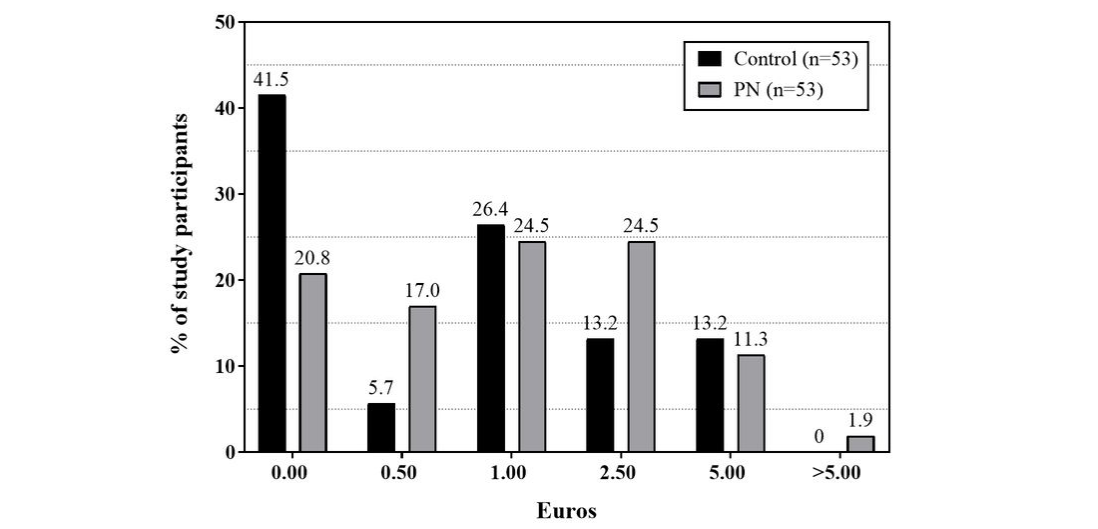
Willingness to pay for the eNutri app across PN and control group.

Participants were additionally asked “What is the maximum you would be willing to pay to purchase the eNutri app for unlimited personal use?” (Figure 3). Across subgroups, 31.1% (total: 33/106; control: 22/53, 42%; PN: 11/53, 21%; *P*=.04) would not pay anything at all to purchase the eNutri app, whereas 55.7% (59/106) would pay between €0.50 (US $0.53) and €2.50 (US $2.67). In total, 13.2% (14/106) would pay €5.00 (US $5.34) or more. Overall willingness to pay did not significantly differ between the PN group and the control group (*P*=.3).

Open-ended feedback, suggestions, or problems encountered during the eNutri2019 study were collected from 84 participants (control: 45/53, 85%; PN: 39/53, 74%). A categorization was made into 3 main topics: app in general, FFQ, and dietary recommendations.

Respondents positively highlighted the high intuitiveness of the eNutri app in general, coupled with good comprehensibility and ease of use (total: 22/106, 20.8%; control: 10/53, 19%; PN: 12/53, 23%). In terms of the FFQ, the time-consuming process of data entry was remarked as too long and extensive (total: 12/106, 11.3%; control: 8/53, 15%; PN: 4/53, 8%). Many participants found it difficult to remember the foods and their quantities they had eaten in the previous month and to enter them in the correct section of the FFQ (total: 18/75, 24%; control: 6/40, 15%; PN: 12/35, 34%). At the same time, 12% (9/75) of participants noted positively the photos of the food in different portion sizes included in the FFQ and considered them as very helpful in answering the survey questions. A rather small percentage (7/106, 6.6%) of the responders (control: 3/53, 6%; PN: 4/53, 8%) suggested a superordinate categorization into food groups (vegetables, meat, etc.), as this would have made it easier to keep track and thus to answer the FFQ. Regarding the dietary recommendations, 45.3% (48/106) of the study participants gave feedback. Nutritional advice in the PN and control groups should be considered in a differentiated manner. In the control group, 58% (31/53) indicated that the nutrition report was rather impersonal, superficial, and too general, yet contained good, easy-to-understand nutrition tips; however, they lacked helpful information for the implementation into everyday life. In the PN group, conversely, 32% (17/53) stated that they would have liked a more detailed nutrition report including more specific information about the amounts of foods to be increased; 16% (17/106) of all respondents (control: 11/53, 21%; PN: 6/53, 11%) reported that the dietary recommendations were not tailored to their individual diet or were incorrect according to the information indicated in the FFQ (eg, no meat consumption as a vegan followed by a recommendation to eat meat).

## Discussion

### Principal Findings

One of the objectives of the eNutri2019 pilot study as part of the EIT Food Quisper project was to introduce the German eNutri app and evaluate its usability metrics in a real-life setting. From a secondary perspective, this study sought to assess whether the provision of personalized dietary recommendations by the eNutri app provides greater benefits related to nutrition behavior changes than generic dietary advice.

Analysis of the system’s usability yielded an overall mean score of 78.4, indicating that using the eNutri app is clearly above an average experience (SUS 68) [29]. As no statistically significant differences between groups could be identified, a good performance across all users can be assumed. Ferrara et al [30] reviewed diet-tracking apps and, inter alia, evaluated the usability of the top 7 diet-tracking apps found within the most popular online app stores in 2017. Across all apps considered, a mean value of 71 (range 46.7-89.2) was obtained. In comparison, the eNutri app achieved a similar usability SUS score, putting it on par. As an indicator for technical and perceived usability performance in terms of effectiveness, efficiency, ease of use, and user satisfaction, the high SUS score of eNutri stands proxy for high user acceptability and great usability.

Within app stores, most nutrition-related apps are free of charge to download but include additional fees to purchase a premium version to get access to more features. The price of an app can be seen as one key criterion for selecting an app within the app stores [31]. One-third of the eNutri2019 study participants stated that they would not pay anything at all to purchase the eNutri app. More than half would pay between €0.50 (US $0.53) and €2.50 (US $2.67). These results are in line with qualitative and quantitative research, showing a tendency toward a reluctant willingness to pay for nutrition apps [32,33].

Another aspect showing the value of an app is the common star rating. Overall, participants rated the app with 3.3 out of 5 stars on average, with no significant differences between the PN and the control group. Among other popular nutrition apps, such as MyFitnessPal, Lifesum, or Freeletics Nutrition, 72% hold an average rating between 4 and 4.5 stars [34], indicating the need to further improve the eNutri app. Ratings are a reflection of user experience that involves a multidimensional interplay of system usability, context of use, expectations, perceived utility, and emotions before and after using an app [35]; thus, a rating of 3.3 stars implies that eNutri exhibits likely shortcomings in these areas that require further investigation and remediation.

A significant factor in this context is also the FFQ. FFQs are commonly integrated within diet-tracking apps to assess dietary behavior. Depending on the number of food items included, the completion time varies between 30 and 60 minutes [36]. The completion time of an FFQ is positively correlated with the potential to create typical biases [37], therefore a shorter completion time is desirable. The mean completion time for the eNutri app was 26.7 minutes, with no significant differences between groups. Franco et al [16] reported a mean completion time of 22.9 minutes in their formative study on the UK version of the eNutri app. Although the completion time of the eNutri FFQ is within a comparable range, its length was burdensome for respondents, as reflected in the open-ended feedback. To decrease the effort for the user, investigations on how to streamline the FFQ without reducing its quality need to be undertaken.

### Comparison With Prior Work

Regarding the potential of the eNutri app to change a users’ dietary behavior, the majority of the participants stated via open feedback that using the app motivated them to make changes in their diets and improved their awareness of healthy eating behavior. The eNutri app was positively evaluated by users, stating that the process of monitoring their dietary intake initiated a self-reflecting process, rising their awareness about unbalanced and unhealthy dietary habits. This process has also been described in various theories on behavior change, identifying *consciousness raising* as a crucial point in the process of change [38]. Furthermore, studies demonstrated a positive effect of food intake recording on the awareness of food quality and quantity [39]. This is in line with previous research indicating that perceived app utility and personalization positively affect the continuance usage intention [39-41]. Participant’s feedback suggests that the personalized dietary recommendations tended to being more effective than the generic recommendations, at least in providing nutrition knowledge and support for targeted dietary adjustments tailored to an individual and his/her FFQ-derived food preferences.

### Strengths and Limitations

The purpose of this study was to assess the usability of the German version of the eNutri app. Therefore, the design of a pilot study was applied.

First, the implementation of the validated and well-established graphical FFQ strengthens the design of the study. An FFQ is a commonly used dietary assessment instrument that provides information on the type, frequency, and quantity of foods consumed and allows for population estimates. However, FFQs are prone to over- and underreporting due to their retrospective character and the time-consuming and demanding nature. By contrast, the application of prospective dietary assessment methods reduces recall bias and shows higher validity and precision, such as a food diary with a higher validity and precision [42]. The application of a prospective instrument such as a food diary should therefore be reconsidered.

Second, participants’ open feedback revealed a high burden due to the long completion time. At the same point, participants’ responses showed that the more personalized a dietary recommendation was, the better it tended to be accepted by them. This highlights the importance of collecting detailed food intake information via the FFQ, to enable the system to detect individual consumption patterns and preferences to subsequently provide tailored recommendations. Therefore, it was essential that the same foods in different processing stages were repeatedly queried in the FFQ (eg, vegetables in raw or cooked condition), because changes in food texture and consistency can greatly influence personal food preferences. For instance, it does not necessarily mean that if someone likes to eat raw vegetables, which can be quite crunchy in consistency, this person will also like them in cooked form with more smoothness. In return, this also means that if the FFQ was shortened, relevant information about food preferences cannot be obtained. A detailed and comprehensive FFQ, by contrast, improves the dietary recommendations, but the temporary burden to fill in the high number of items poses the risk of dropouts. This results in a certain dichotomy, and thus a balance between information demands and effort for completion needs to be found.

Third, evidence suggests that females tend to underreport in nutritional surveys [43]. Overall, there was a high proportion of women among the eNutri study population. This may be due to several reasons, one of which is the observation that women can be quite successfully recruited via social media (eg, Twitter, Facebook) [44], which was also the primary recruitment channel in the eNutri2019 study. Further, gender differences in health information–seeking behavior are known, just as a higher motivation and interest of females to deal with health-related information, coupled with a higher consciousness of nutrition [45,46]. Therefore, we will redesign our recruitment materials to appeal to a broader spectrum of the population for a follow-up study.

Fourth, an important issue arising from the eNutri pilot study is the focus on a healthy adult population. Thus, dietary recommendations provided by the eNutri app are tailored to this target group, while people with special diets requirements (eg, food intolerances, allergies) or certain nutrition-related diseases are excluded. To make the app more compatible for a broader and heterogeneous target audience, it should be adapted to diverse (nutritional) needs, taking into account not only anthropometric, physical characteristics, and individual food preferences, but also medical and behavioral traits or even genetic factors.

Fifth, the main aim was to assess the usability of the German eNutri app. Results revealed that the tool is appropriate for different user groups. The focus of the differentiation between the PN and control groups was concentrated on the open feedback. To perform an in-depth comparison of advice given to the PN and control groups, further studies with longer study periods are needed.

### Further Directions

To effectively adopt positive dietary changes, a long-term implementation of the recommendations into daily habits is essential. Considering that many study participants reported having difficulties in implementing the received dietary recommendations, additional features such as a recipe database or cookbook need to be integrated directly into the app. Further improvements in the usability can be achieved by providing some external links to credible databases or scientific literature references or links to the federal Nutrition Society. This is beneficial to expand the app users’ knowledge on food and nutrition, if desired. It has also been shown that coaching sessions as well as web-based coaching classes positively influence the success of dietary interventions [47]. The integration of features enabling more personal guidance (eg, chat or video coaching) within the eNutri app is another suggestion for improvement. Some nutrition apps were already designed integrating chatbot functions to support weight loss interventions [48]. To boost long-term user engagement, more in-app personalization, for example, features enabling personal goal setting and progress tracking [49], or integration of personal avatars, is advisable. Incorporating gamification elements to better communicate nutrition knowledge and raise awareness about healthier food choices is also an option with proven benefits [50].

### Conclusions

The eNutri app is a field-tested feasible and usable web tool to assess habitual dietary intake. The eNutri app is unique in that it is specifically tailored to the eating habits of a Western European population, more precisely a German population. It takes country-specific food items and eating habits into account and was developed by nutrition experts. The image-based validated food quantification and the app’s ease of use contributed positively to an above average usability experience of the eNutri2019 study participants. Users’ experience on the eNutri app’s usability was above average across different user groups. A higher level of personalization within nutritional recommendation was seen as more supportive for the implementation of positive dietary changes, in the short as well as in the long term. However, the eNutri app needs to be further adapted and extensions regarding behavioral change features need to be included. In total, the German eNutri app represents a promising tool for assessing habitual dietary intake and could become a valuable instrument to support the accomplishment of healthy dietary habits within a wide spectrum of different user groups. As a time- and cost-effective tool, it has the potential to alleviate the burden of diet-related diseases on the health care system.

## Appendix

Multimedia Appendix 1 Flowchart of study participants' recruitment.

## Abbreviations

BLS: German Food Code and Nutrient Database
DGE: German Nutrition Society
DQS: Diet Quality Score
EPIC: European Prospective Investigation into Cancer
FFQ: food frequency questionnaire
ICT: information and communications technology
PN: personalized nutrition
SUS: System Usability Scale
t1: time point 1
t2: time point 2
