# Supplementary figures and images for: The Assessment of a Personalized Nutrition Tool (eNutri) in Germany: Pilot Study on Usability Metrics and Users’ Experiences
Source: JMIR Form Res. 2022 Aug 4;6(8):e34497. doi: 10.2196/34497 (PMC9389388; doi:10.2196/34497)

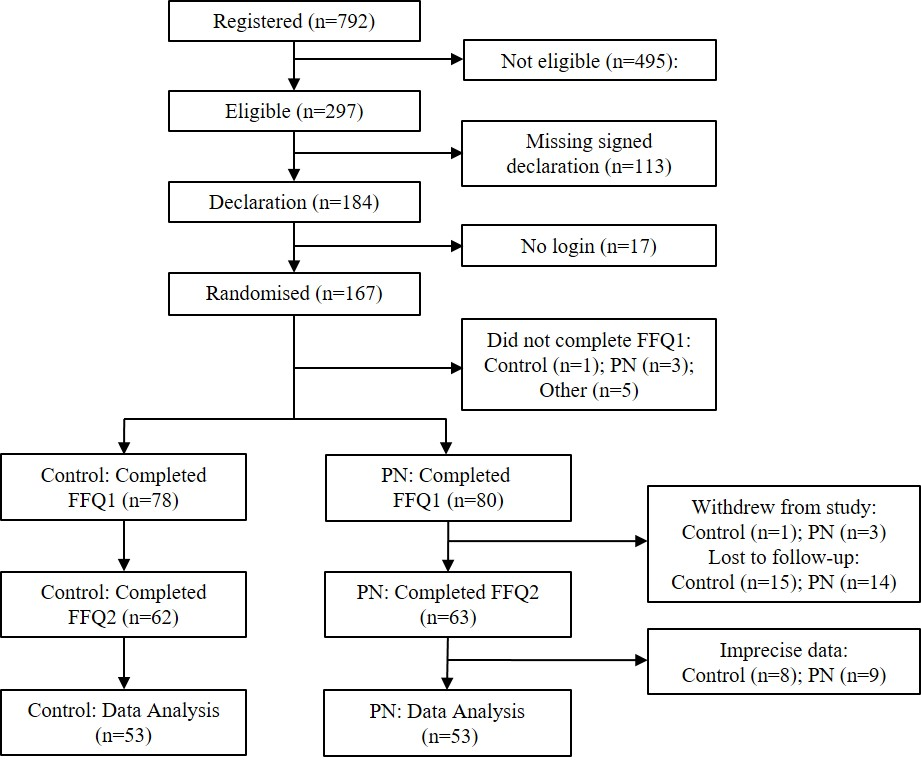

Supplement: Multimedia Appendix 1 [file formative_v6i8e34497_app1.png]
